# Evaluation of deformity correction and complications using sublaminar band anchoring in the primary curve of patients with adolescent idiopathic scoliosis

**DOI:** 10.1186/s12891-025-08908-1

**Published:** 2025-07-04

**Authors:** Stefan Hemmer, Moritz von Falkenhayn, Raphael Trefzer, Lukas Baumann, Wojciech Pepke

**Affiliations:** 1 Clinic for Orthopaedics, Heidelberg University Hospital, Schlierbacher Landstrasse 200a, Heidelberg, 69118 Germany; 2 Institute of Medical Biometry, University of Heidelberg, Heidelberg, Germany

**Keywords:** Adolescent idiopathic scoliosis, Sublaminar bands, Pedicle screws, Posterior spinal fusion

## Abstract

**Background:**

Adolescent idiopathic scoliosis (AIS) is a complex three-dimensional spinal deformity requiring surgical intervention in severe cases. Posterior spinal fusion (PSF) with pedicle screws is the gold standard for correction; however, rigid curves and significant rotational deformities may benefit from adjunctive fixation methods. Sublaminar bands have been proposed to enhance deformity correction while potentially reducing complications. This study aims to evaluate the impact of sublaminar band-assisted constructs versus pedicle screw-only constructs on deformity correction and complication rates in AIS surgery.

**Methods:**

A retrospective cohort study was conducted on 165 AIS patients treated with PSF between 2010 and 2024. Patients were divided into two groups: pedicle screws only (*n* = 124) and pedicle screws with sublaminar bands (*n* = 41). Demographic, intraoperative, and radiographic data were collected. Propensity score matching was performed to reduce selection bias, and statistical analysis included t-tests, Mann-Whitney U-tests, and Chi-square tests.

**Results:**

The mean preoperative Cobb angle was comparable between groups (*p* > 0.05). While coronal and sagittal correction outcomes were similar, the sublaminar band group showed superior correction in axial rotation (Raimondi angle improvement: 9.8° vs. 5.9°, *p* = 0.015). No significant differences were observed in overall complication rates or revision surgeries (*p* > 0.05). Neurological complications were rare in both groups, with intraoperative neuromonitoring contributing to safety.

**Conclusion:**

Sublaminar band-assisted constructs provide enhanced axial rotation correction in AIS surgery without increasing complication rates. These findings support the use of sublaminar bands as an effective adjunct to pedicle screws, particularly in cases involving rigid or rotational deformities. Further multicenter prospective studies are warranted to confirm these results.

## Introduction

Adolescent idiopathic scoliosis (AIS) is a three-dimensional deformity of the spine characterized by lateral curvature and vertebral rotation, with an incidence of 1–3% among adolescents [1]. Surgical intervention is often recommended for curves exceeding 40° to prevent further progression and achieve optimal spinal alignment. Posterior spinal fusion (PSF) with pedicle screw constructs has become the gold standard for surgical correction due to its biomechanical stability and capacity to achieve substantial correction in all planes [2]. However, rigid spinal deformities or those with significant rotational components present unique challenges in achieving optimal correction and maintaining stability postoperatively [3].

Sublaminar bands, used as supplementary fixation to pedicle screws, have emerged as a potential solution for enhancing deformity correction in patients with rigid or highly rotational curves [4]. The technique involves passing flexible polyethylene bands beneath the lamina and anchoring them to a rod, providing both axial derotation and additional correctional forces [5]. Unlike traditional hooks or wires, sublaminar bands distribute loads over a broader area, potentially reducing the risk of laminar fractures while achieving enhanced stability [5]. Despite its theoretical advantages, concerns regarding neurological safety, intraoperative complexity, and postoperative complications remain key areas of investigation.

To date, literature evaluating the efficacy and safety of sublaminar bands in AIS surgery remains limited, with mixed outcomes regarding their superiority over pedicle screw-only constructs. Therefore, this study aims [1] to analyze the impact of sublaminar band-assisted constructs compared to conventional pedicle screw constructs on radiographic correction in all planes and [2] postoperative complication rates. By performing a comprehensive retrospective review of patients undergoing posterior fusion for AIS at a tertiary care center, this study seeks to provide valuable insights into the role of sublaminar bands in modern scoliosis surgery.

## Methods

### Study cohort

**Fig. 1:**
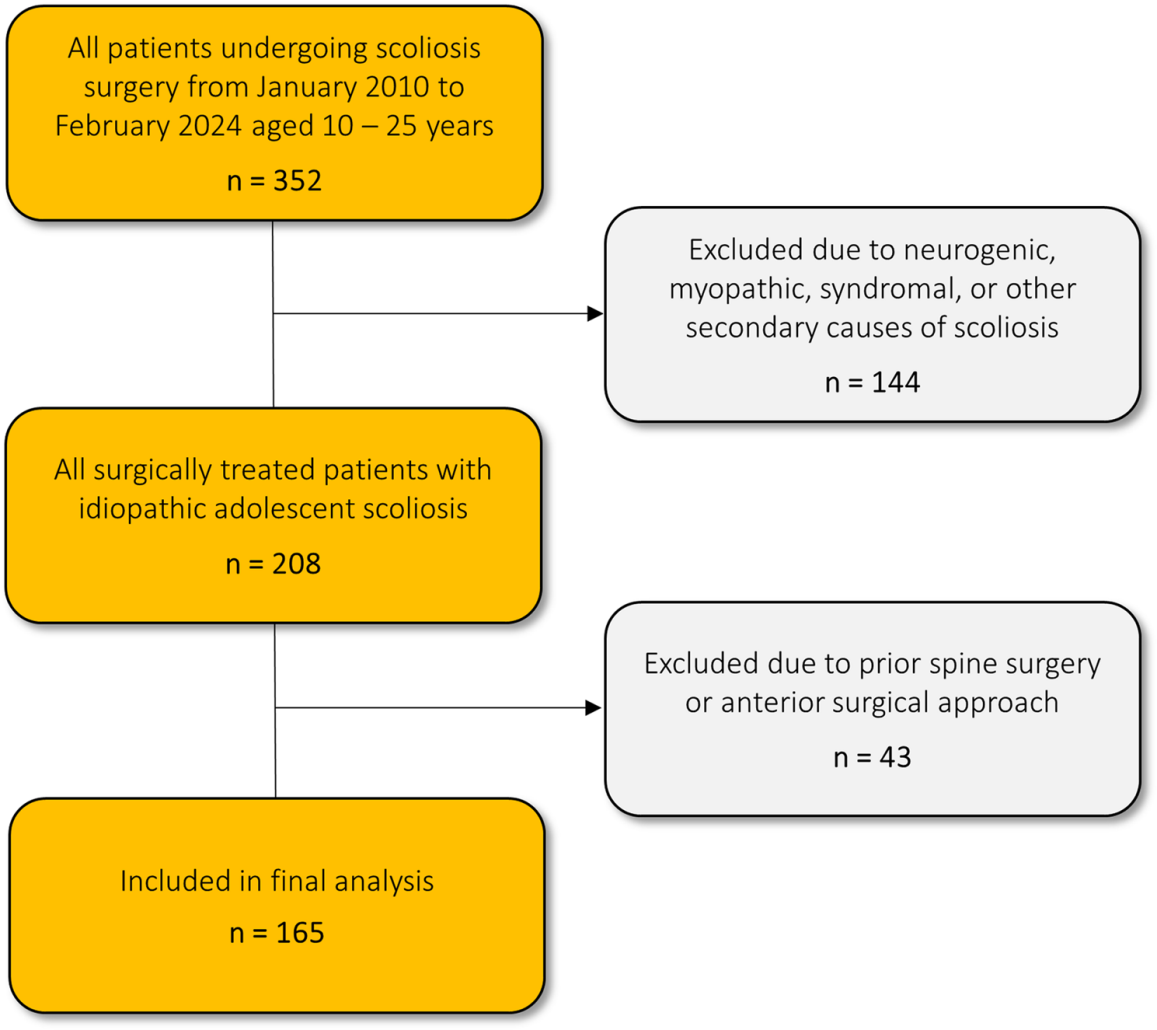
Diagram depicting the process of inclusion and exclusion criteria for the study population

This study presents a retrospective, single-center, multi-surgeon cohort analysis involving a consecutive series of 165 patients who underwent posterior spinal fusion (PSF) for AIS from January 2010 to February 2024. The follow-up duration was clearly documented, and final radiographic outcomes were analyzed. The inclusion criteria encompassed patients aged 10 to 25 years at the time of surgery and all Lenke classification types. Exclusion criteria included neurogenic, myopathic, syndromal, or other secondary causes of scoliosis, prior spine surgery as well as anterior surgical approach. The inclusion and exclusion process is illustrated in Fig. 1. Ethical approval for the study was granted by the ethics committee of the medical faculty of Heidelberg University (approval No. S-344/2024). In light of the retrospective nature of this study, which involved the utilization of anonymized data derived exclusively from preexisting clinical records, patient informed consent was deemed unnecessary. The analysis of the data was conducted solely by the treating physicians who possessed the requisite authorization for data access.

### Surgical technique

**Fig. 2:**
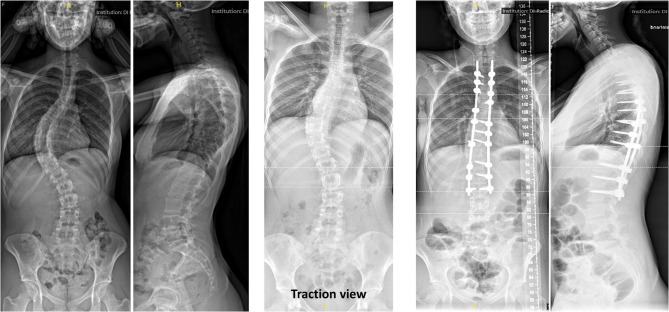
Pre- and postoperative A.P. and lateral radiographs of a 16-year-old female patient with idiopathic adolescent scoliosis, treated with posterior spinal fusion using pedicle screws only

**Fig. 3:**
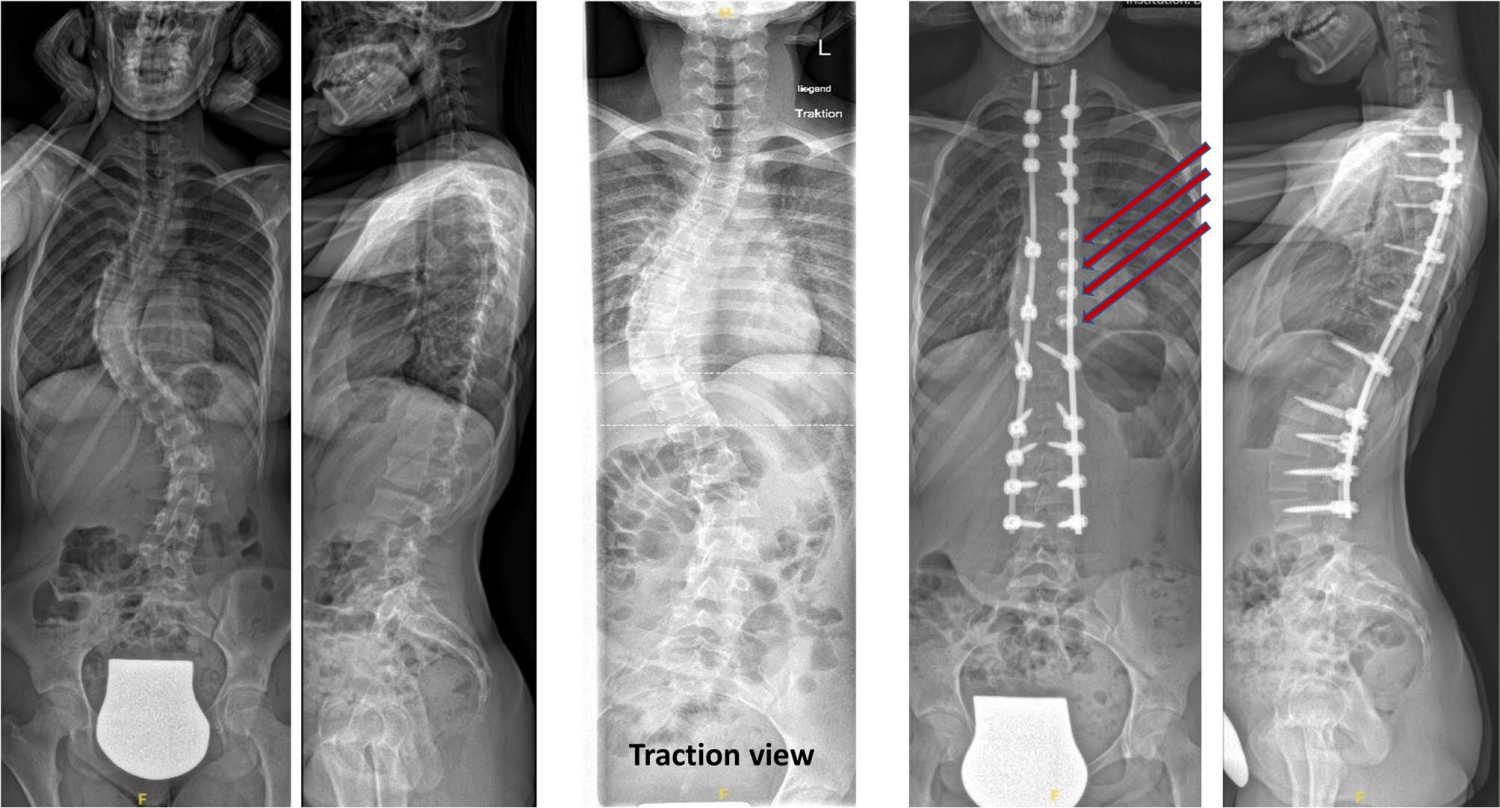
Pre- and postoperative A.P. and lateral radiographs of a 14-year-old female patient with idiopathic adolescent scoliosis, treated with posterior spinal fusion and the additional use of four sublaminar bands applied at the apex of the main curve

All surgeries were conducted by two experienced senior spine surgeons at a single tertiary care hospital. Surgery was performed in a prone position and under general anesthesia. Intraoperative neuromonitoring with a triggered electromyogram device for measurement of sensory and motor evoked potentials was used in all patients. Posterior pedicle screw fixation was employed in all patients (Fig. 2), while a subset received supplementary fixation with sublaminar bands (Fig. 3). Indications for the use of sublaminar bands included rigid main curves on traction/bending radiographs and/or significant rotational deformity of the main curve defined as Raimondi angles exceeding 25°. Ponte osteotomies were performed in cases with rigid deformities to facilitate greater correction. Pedicle screw derotation maneuvers were routinely performed in both study groups to address the rotational component of the spinal deformity; additionally, in the sublaminar band group, bands were also utilized to enhance the derotation effect. To promote bony fusion, autologous bone graft was harvested from the spinous processes and distributed over the fusion site.

### Data collection and analysis

Demographic data (including sex, age, height, weight, body mass index (BMI), and Lenke classification) were collected from patient records. Additionally, perioperative and intraoperative metrics such as operative time, blood loss, the necessity for transfusion of allogenic or autologous blood components, complications, and revision surgeries were documented.

Preoperatively, each patient received a comprehensive radiologic assessment, which included standing anteroposterior (A.P.) and lateral radiographs, as well as supine traction AP radiographs of the entire spine and fulcrum side bending radiographs targeting the main curve. Traction radiographs were obtained with the patient in a supine position, where traction was applied at the neck using a submandibular grip by an experienced physician. For bending radiographs patients were positioned on their convex side with a fulcrum placed beneath the apex of the spinal curve. Routine postoperative follow-up involved standing AP and lateral whole spine views.

For radiographic analysis, calibrated DICOM digital images were evaluated with Surgimap for Mac (Surgimap^®^ Version 2.3.2.1; Nemaris Inc., New York, NY, USA). An orthopaedic surgery resident (MF) conducted all measurements, while an attending spine surgeon (WP) randomly selected 50 patients and independently obtained all metrics again. Subsequently, interrater reliability was established. Coronal parameters included the main, secondary, and tertiary curve Cobb angles, as well as rotational deformity evaluated via the Raimondi angle [6]. Furthermore, curve flexibility was determined by the relative and absolute differences in Cobb angle between standing X-rays and traction/bending views. Sagittal parameters encompassed lumbar lordosis, thoracic kyphosis (TK) (T2-T5, T5-T12, and T1-T12), and spinopelvic parameters, including pelvic incidence, pelvic tilt, and sacral slope.

### Statistical analysis

Statistical analysis was performed using SPSS for Windows (Version 29.0; IBM, Armonk, NY, USA). Descriptive statistics included the arithmetic mean, standard deviation, minimum, and maximum values. Data were assessed for normal distribution using the Kolmogorov-Smirnov test with Lilliefors correction (sample size > 50) or the Shapiro-Wilk test (sample size < 50). Based on the distribution results, a paired and unpaired two-sided t-test or a Mann-Whitney U-test was applied as appropriate. For complication analysis, a Pearson Chi-Squared test was employed. For analyzing the Lenke classification, Fisher’s exact test was used. For additional analysis of coronal parameters, manual propensity score matching (PSM) was performed Matching was performed using a binary logistic regression model with a tolerance of 0.05, where we included key preoperative variables: the Cobb angles of the main and secondary curves, as well as the preoperative rotational deformity assessed by the Raimondi angle, resulting in two groups of 32 patients in each. These variables were selected based on their relevance in predicting surgical outcomes and their role in defining the severity of the deformity. To ensure balance between the groups, we performed a manual matching procedure, which aimed to create two comparable groups in terms of these critical parameters. Post-matching balance was assessed through standardized mean differences, confirming the comparability of the groups. Statistical significance was determined by a *p*-value of < 0.05 for all tests.

## Results

### Global analysis

This study evaluated the outcomes of AIS 165 patients who posterior fusion at our institution between 2010 and 2024, focusing on coronal, sagittal and axial profile correction, as well as postoperative complications. The mean age of patients at the time of the surgery was 16 years (range: 11–25 years). 124 patients were treated with posterior pedicle screw fixation only, while 41 patients received additional sublaminar band implantation. Statistical comparisons between the subgroups revealed no significant differences (*p* > 0.05) in terms of sex and age at index surgery, BMI and instrumented segments. Furthermore, the mean final follow-up duration was 18 months in the sublaminar bands group and 35 months in the pedicle screw-only group. Baseline demographic patient characteristics at surgery are depicted on Table 1.

**Table 1:** Patient characteristics from the medical records

	Sublaminar bands	Pedicle screws only	*p*-value
n	41	124	-
Female (%)	73.2	75.6	0.756
Age (years; mean, SD)	16.0 (2.8)	16.2 (2.7)	0.987
Weight (kg; mean, SD)	59.2 (13.2)	62.1 (17.1)	0.586
Height (cm; mean, SD)	165.0 (10.3)	167.6 (8.8)	0.291
BMI (kg/m^2^; mean, SD)	21.7 (3.8)	22.0 (5.6)	0.727
Instrumented segments (n; mean, SD)	11.5 (1.5)	10.8 (2.1)	0.074
Lenke 1	31	79	
Lenke 2	0	4	
Lenke 3	6	10	
Lenke 4	0	0	
Lenke 5	3	19	
Lenke 6	1	12	
Operation time (min; mean, SD)	257 (64)	255 (105)	0.235
Blood loss (ml; mean, SD)	1102 (997)	1258 (1131)	0.301

*SD* Standard deviation

### Impact analysis of sublaminar bands/pedicle-screws constructs in comparison to pedicle-screws only constructs on coronal and axial correction

Pre- and postoperative radiographs were available for all 165 patients. The mean preoperative main curve Cobb angle (MC Cobb) in the pedicle screw only group was 58.2°, while it was 62.8° in the group with supplemental sublaminar bands (*p* = 0.09). There was no significant difference in preoperative average secondary curve Cobb angle (SC Cobb) (41.7° vs. 41.2°; *p* = 0.828). However, the main curve rotation (Raimondi 1) was significantly greater in the sublaminar band group (29.7° vs. 25.9°; *p* = 0.042). Main curve flexibility, as assessed by traction radiographs, was similar between groups, but the group treated with pedicle screws only demonstrated a less rigid secondary curve (29.7° vs. 15.4°; *p* < 0.001). There were no significant differences in postoperative main and secondary Cobb angles. However, the correction in main curve rotation was significantly greater in the group treated with sublaminar bands (9.8° vs. 5.9°; *p* = 0.015). After propensity score matching (*n* = 32), no significant difference in rotational correction was observed. The smaller sample size of the sublaminar band group (*n* = 41) compared to the pedicle screw-only group (*n* = 124) is acknowledged as a limitation potentially affecting generalizability. All pre- and postoperative radiographic data, both with and without propensity score matching, are presented in Tables 2 and 3.

**Table 2:** Pre- and postoperative data of coronal and axial parameters for both study groups

Preoperative coronal parameters			
	Sublaminar bands	Pedicle screws only	*p*-value
MC Cobb angle (°)	62.8 (16.2)	58.2 (12.9)	0.09
SC Cobb angle (°)	41.2 (10.8)	41.7 (10.3)	0.828
MC Raimondi angle (°)	29.7 (10.8)	25.9 (10.7)	0.042*
SC Raimondi angle (°)	14.1 (11.8)	19.6 (12.6)	0.025*
MC flexibility (°)	24.6 (9.7)	22.9 (7.8)	0.314
MC relative flexibility (%)	0.380 (0.119)	0.408 (0.134)	0.285
SC flexibility (°)	15.4 (7.6)	29.7 (9.2)	<0.001*
SC relative flexibility (%)	0.361 (0.140)	0.525 (0.138)	<0.001*

Postoperative coronal parameters			
	Sublaminar bands	Pedicle screws only	*p*-value
MC Cobb angle (°)	24.4 (7.7)	24.6 (8.1)	0.917
SC Cobb angle (°)	19.7 (6.9)	21.4 (8.1)	0.186
MC Raimondi angle (°)	19.9 (10.9)	19.7 (10.5)	0.929
SC Raimondi angle (°)	10.4 (8.5)	13.0 (9.5)	0.146
MC Cobb angle absolute difference (°)	38.4 (11.3)	33.7 (9.8)	0.011
MC Cobb angle relative difference (%)	0.609 (0.085)	0.570 (0.117)	0.054
SC Cobb angle absolute difference (°)	21.6 (10.0)	20.6 (8.0)	0.599
SC Cobb angle relative difference (%)	0.509 (0.157)	0.490 (0.148)	0.486
MC Raimondi angle absolute difference (°)	9.8 (8.6)	5.9 (7.9)	0.015*
SC Raimondi angle absolute difference (°)	4.4 (7.9)	6.7 (8.2)	0.228

Mean (standard deviation) given

*MC* Main curve, *SC* Secondary curve, *TC* Tertiary curve

**Table 3:** Pre- and postoperative data of coronal and axial parameters for both study groups after propensity-score matching

Preoperative coronal parameters after propensity score matching (*n* per group = 32)			
	Sublaminar bands	Pedicle screws only	*p*-value
MC Cobb angle (°)	61.5 (16.9)	62.2 (13.6)	0.524
SC Cobb angle (°)	41.6 (10.4)	43.4 (10.5)	0.702
MC Raimondi angle (°)	27.8 (11.3)	27.8 (9.8)	0.995
SC Raimondi angle (°)	13.6 (11.5)	15.5 (11.5)	0.381
MC flexibility (°)	23.2 (9.8)	23.4 (7.0)	0.932
MC relative flexibility (%)	0.367 (0.124)	0.386 (0.130)	0.624
SC flexibility (°)	15.8 (7.5)	31.1 (10.5)	<0.001*
SC relative flexibility (%)	0.373 (0.139)	0.492 (0.125)	0.005*

Postoperative coronal parameters after propensity score matching (*n* per group = 32)			
	Sublaminar bands	Pedicle screws only	*p*-value
MC Cobb angle (°)	24.2 (8.3)	26.8 (7.1)	0.063
SC Cobb angle (°)	20.3 (7.5)	21.4 (6.6)	0.259
MC Raimondi angle (°)	19.5 (10.1)	20.9 (9.1)	0.480
SC Raimondi angle (°)	9.8 (8.0)	10.9 (8.0)	0.459
MC Cobb angle absolute difference (°)	37.3 (11.7)	35.6 (9.4)	0.638
MC Cobb angle relative difference (%)	0.605 (0.091)	0.561 (0.083)	0.048*
SC Cobb angle absolute difference (°)	21.3 (9.0)	21.0 (7.1)	0.810
SC Cobb angle relative difference (%)	0.505 (0.155)	0.486 (0.126)	0.394
MC Raimondi angle absolute difference (°)	8.3 (7.0)	6.9 (5.3)	0.337
SC Raimondi angle absolute difference (°)	3.8 (7.3)	4.6 (7.6)	0.685

Mean (standard deviation) given

*MC* Main curve, *SC* Secondary curve, *TC* Tertiary curve

### Impact analysis of sublaminar bands/pedicle-screws constructs in comparison to pedicle-screws only constructs on sagittal alignment

Analysis of sagittal radiographic parameters revealed no significant differences between groups before or after surgery (Table 4). In both groups, LL and TK (T2-T5 and T1-T12) was improved postoperatively (Table 5).

**Table 4:** Pre- and postoperative data of sagittal parameters for both study groups

Preoperative sagittal parameters			
	Sublaminar bands	Pedicle screws only	*p*-value
Lumbar lordosis (°)	57.5 (13.4)	56.6 (11.6)	0.905
Pelvic incidence (°)	52.1 (12.5)	51.6 (10.6)	0.923
Pelvic tilt (°)	10.7 (8.0)	11.3 (6.3)	0.953
Sacral slope (°)	41.4 (10.1)	40.3 (8.7)	0.868
Thoracic kyphosis T2-T5 (°)	11.8 (7.1)	13.3 (8.7)	0.158
Thoracic kyphosis T5-T12 (°)	22.1 (14.6)	26.0 (14.2)	0.225
Thoracic kyphosis T1-T12 (°)	31.9 (14.9)	36.8 (14.7)	0.097

Postoperative sagittal parameters			
	Sublaminar bands	Pedicle screws only	*p*-value
Lumbar lordosis (°)	51.4 (10.6)	51.3 (11.3)	0.999
Pelvic incidence (°)	52.0 (12.9)	51.3 (10.5)	0.649
Pelvic tilt (°)	12.1 (10.2)	12.0 (7.8)	0.868
Sacral slope (°)	39.9 (9.3)	39.3 (8.5)	0.666
Thoracic kyphosis T2-T5 (°)	15.6 (5.9)	17.1 (6.8)	0.135
Thoracic kyphosis T5-T12 (°)	19.4 (8.1)	21.5 (9.2)	0.433
Thoracic kyphosis T1-T12 (°)	35.6 (9.9)	38.4 (10.0)	0.203

**Table 5:** Analysis of sagittal parameters from preoperative to postoperative status for both study groups

Intragroup comparison of sagittal parameter before and after surgery (sublaminar band group)			
	Preoperatively	Postoperatively	*p*-value
Lumbar lordosis (°)	56.5 (13.1)	51.5 (10.6)	0.002*
Pelvic incidence (°)	52.1 (12.2)	52.0 (12.9)	0.892
Pelvic tilt (°)	10.8 (7.5)	12.1 (10.2)	0.269
Sacral slope (°)	41.4 (10.1)	39.9 (9.3)	0.185
Thoracic kyphosis T2-T5 (°)	11.4 (6.8)	15.3 (5.8)	<0.001*
Thoracic kyphosis T5-T12 (°)	22.6 (13.6)	20.4 (8.5)	0.201
Thoracic kyphosis T1-T12 (°)	31.9 (14.3)	36.2 (10.2)	0.019*

Intragroup comparison of sagittal parameter before and after surgery (pedicle screw only group)			
	Preoperatively	Postoperatively	*p*-value
Lumbar lordosis (°)	56.6 (12.1)	51.6 (11.3)	<0.001*
Pelvic incidence (°)	51.6 (11.4)	51.3 (11.2)	0.410
Pelvic tilt (°)	11.3 (6.7)	12.0 (8.3)	0.240
Sacral slope (°)	40.3 (8.8)	39.3 (8.7)	0.105
Thoracic kyphosis T2-T5 (°)	13.3 (8.3)	17.0 (6.7)	<0.001*
Thoracic kyphosis T5-T12 (°)	25.3 (14.4)	21.4 (9.0)	<0.001*
Thoracic kyphosis T1-T12 (°)	36.4 (14.2)	38.5 (9.9)	0.047*

### Analysis of the parameters at final follow-up

Follow-up measurements revealed several interesting changes in the coronal parameters over time. The MC Cobb angle showed a slight but significant increase at final follow-up in the sublaminar bands group (MC Cobb post-op vs. final follow-up: 24.4° vs. 26.4°; *p* = 0.030), whereas no significant change was observed in the pedicle screw only group (MC Cobb post-op vs. final follow-up: 24.6° vs. 25.9°; *p* = 0.072). No significant changes over time were observed in the SC Cobb angle in either group. At final follow-up, both the MC and SC Raimondi angles showed a slight but significant change in the pedicle screws only group (MC Raimondi: 19.7° vs. 21.9°; *p* < 0.001, and SC Raimondi: 13.0° vs. 12.1°; *p* = 0.040). In the sublaminar bands group, however, there was no significant change in the Raimondi angle at final follow-up.

Regarding sagittal parameters, a relevant increase in both lumbar and thoracic measurements was observed over time. At final follow-up, LL increased significantly in both groups (sublaminar bands group: 51.4° vs. 57.4°; *p* < 0.001, and pedicle screws only group: 51.3° vs. 58.4°; *p* < 0.001).

TK also increased across all measured segments at final follow-up (TK T1-T12: sublaminar bands group: 35.6° vs. 40.2°; *p* < 0.001, pedicle screws only group: 38.4° vs. 42.6°; *p* < 0.001) (TK T5-T12: sublaminar bands group: 19.4° vs. 23.2°; *p* = 0.005, pedicle screws only group: 21.5° vs. 23.1°; *p* < 0.001) (TK T2–T5: sublaminar bands group: 15.6° vs. 17.5°; *p* = 0.018, pedicle screw-only group: 17.1° vs. 19.9°; *p* < 0.001).

With respect to pelvic parameters, corresponding changes were also observed over time. Pelvic tilt (PT) decreased significantly from postoperative to final follow-up in both groups (sublaminar bands group: 12.1° vs. 8.7°; *p* = 0.049, pedicle screw-only group: 12.0° vs. 10.7°; *p* = 0.037). Sacral slope (SS) increased slightly but significantly only in the pedicle screw-only group (SS: 39.3° vs. 41.7°; *p* < 0.001). Pelvic incidence (PI) remained unchanged over time in both groups (*p* > 0.05).

### Analysis of adverse complications after posterior fusion in both groups

Table 6 provides a detailed overview of all complications and revisions. Overall, complications occurred in 28 patients (17%), with superficial impairment of wound healing being the most prevalent. Revision surgery was required in 10.3% of cases, with 52.9% of these procedures consisting of superficial or deep wound debridements. Specific complications, including neurological events and implant-related issues, were documented and managed appropriately, underscoring the safety profile of sublaminar bands. No significant difference according the complications could be detected between both groups.

**Table 6:** Table depicting detailed complications in both groups after surgery

	Sublaminar bands	Pedicle screws only	*p*-value
Complications	5 (12.2%)	21 (16.9%)	0.470
- Wound healing disorder	4	6	
- Late infection	0	4	
- Caudal junctional scoliosis	1	1	
- Proximal junctional kyphosis	0	2	
- Screw loosening	0	1	
- Rod fracture	0	1	
- Painful bursa formation over screw site	0	1	
- Abnormal intraop. neuromonitoring following rod insertion	0	2	
- Beta-2-transferrin detected in drainage fluid	0	2	
- Postop. pneumothorax	0	1	
Revisions	3 (7.3%)	14 (11.3%)	0.468
- Wound revision	3	6	
- Complete implant exchange	0	1	
- Partial implant exchange	0	2	
- Complete implant removal	0	2	
- Partial implant removal	0	1	
- Two-stage rod insertion after abnormal neuromonitoring	0	2	

## Discussion

The findings of this study provide valuable insights into the role of sublaminar bands as an adjunct to pedicle screw constructs in the surgical correction of AIS. Our results demonstrate that sublaminar band-assisted constructs can improve axial rotation correction compared to pedicle screw-only constructs, while maintaining comparable outcomes in terms of coronal and sagittal plane alignment. These findings are consistent with those reported in previous studies evaluating the efficacy of sublaminar bands in AIS correction [7].

Our study found no significant differences in postoperative Cobb angle correction between the sublaminar band group and the pedicle screw-only group. This result aligns with findings from studies by Ilharreborde et al. and Canavese et al., which demonstrated that hybrid constructs using sublaminar bands provide similar coronal plane correction to all-pedicle screw constructs [8, 9]. However, some evidence suggests that sublaminar bands offer specific advantages in restoring thoracic kyphosis, particularly in patients with preoperative hypokyphosis [10, 11]. Despite this theoretical advantage, we observed comparable improvements in thoracic kyphosis and lumbar lordosis in both groups, indicating that both constructs are effective in achieving sagittal plane correction.

A key finding in our study was the superior correction of axial rotation in the sublaminar band group, as measured by the Raimondi angle. This aligns with previous reports highlighting the unique capability of sublaminar bands to apply posteromedial translational forces, thereby enhancing axial derotation [12, 13]. In a study comparing sublaminar bands and pedicle screws, Sikora-Klak et al. reported that sublaminar bands achieved similar levels of apical vertebral derotation compared to differential rod contouring techniques [14]. This suggests that sublaminar bands may be particularly beneficial in cases where rotational deformity is a significant component of the spinal curvature. While this study demonstrates that sublaminar bands provide superior correction of axial rotation, the clinical relevance of this improvement warrants further exploration. Enhanced correction of axial rotation may have significant long-term benefits and potentially improving spinal balance. These improvements could translate into better patient function and quality of life, particularly in patients with rigid or highly rotational curves. Future studies with long-term follow-up are necessary to better understand the full impact of these findings on long-term spinal alignment and patient outcomes.

Concerns regarding the neurological safety of sublaminar bands have been raised due to the proximity of the bands to the spinal cord during insertion [15]. However, our study observed no significant difference in the overall complication rates between the two groups, with neurological deficits being rare. This finding is supported by a large multicenter study by Polirsztok et al., which reported that the incidence of neurological complications associated with sublaminar bands was comparable to that of pedicle screws [12]. Additionally, the use of intraoperative neuromonitoring in all cases likely contributed to the low incidence of neurological events in our cohort.

One potential drawback of sublaminar band-assisted constructs is the increased operative complexity and time. While the results of this study indicate no significant differences in operative time or blood loss between the two groups, the added complexity of using sublaminar bands in scoliosis surgery warrants further consideration. Sublaminar bands may indeed increase surgical difficulty [16], given the additional steps involved in their insertion and the proximity to critical structures such as the dura. However, this study shows that sublaminar bands provide superior correction in axial rotation, especially in cases with rigid or highly rotational curves. This enhanced correction of axial rotation could offer long-term benefits in terms of spinal balance and overall function, potentially justifying the additional complexity for certain patient populations. Therefore, while the operative time and blood loss may be marginally increased, the clinical advantages, particularly in complex deformities, suggest that sublaminar bands are a valuable adjunct to pedicle screws. Larger studies with more extensive data on operative times and patient outcomes would help to further clarify whether the benefits of sublaminar bands outweigh the increased surgical complexity in routine practice.

At final follow-up, both groups demonstrated stable correction with only minor changes over time. In the sublaminar bands group, a slight but statistically significant increase in the main curve Cobb angle was observed, although the secondary curve remained stable. The slight but statistically significant increase in Cobb angle at follow-up in the sublaminar band group could indicate a minor decrease in mechanical stability compared to pedicle screws. However, the increase is minimal and likely clinically insignificant. Therefore, sublaminar bands represent an effective method that contributes meaningfully to deformity correction. Importantly, despite this minor coronal correction loss, sublaminar bands demonstrated superior stability in maintaining axial rotational correction over time. This suggests that while their coronal rigidity may be slightly lower than that of pedicle screws, their ability to preserve rotational alignment makes them a valuable adjunct in three-dimensional deformity correction for AIS. This conclusion is in line with previous research, which has consistently shown that sublaminar bands provide reliable rotational stability and contribute effectively to three-dimensional deformity correction in AIS [14, 15, 17–19]. Sagittal alignment improved progressively in both groups, with significant increases in lumbar lordosis and thoracic kyphosis. Additionally, favorable changes in pelvic parameters, including a reduction in pelvic tilt, were observed, while pelvic incidence remained stable. These findings suggest that both constructs provided durable deformity correction, with minor expected adaptations during postoperative spinal remodeling. These results are in line with the meta-analysis by Pasha et al., as both demonstrate significant changes in sagittal alignment, including increases in lumbar lordosis and thoracic kyphosis, with minor long-term adaptations in pelvic parameters following posterior spinal fusion in adolescent idiopathic scoliosis patients [20].

Our study has several limitations, including its retrospective design and single-center setting, which may limit the generalizability of the findings. Important limitation of this study is the relatively small sample size in the sublaminar band group (*n* = 41) compared to the pedicle screw-only group (*n* = 124). While the statistical analysis conducted is robust, the smaller sample size in the sublaminar band group may impact the generalizability of the findings. Although the retrospective design of this study has inherent limitations, the use of PSM is an effective strategy to mitigate selection bias. Nevertheless, residual confounding factors may still influence the results due to the non-randomized nature of the study. While the use of PSM reduces selection bias, we acknowledge that residual confounding may still exist due to the non-randomized nature of this retrospective study. Future prospective, multicenter studies with randomized designs are needed to further validate these findings and reduce the impact of potential confounders. These studies could provide more robust evidence and enhance the generalizability of the results.

The results of this study suggest that sublaminar bands can serve as a valuable adjunct to pedicle screws in AIS surgery, particularly for patients with rigid or highly rotational curves. The improved correction of axial rotation may have long-term implications for spinal balance and function. Furthermore, the comparable complication rates and radiographic outcomes indicate that sublaminar bands can be used safely in conjunction with pedicle screws, provided that appropriate surgical technique and intraoperative monitoring are employed.

## Conclusion

In conclusion, the addition of sublaminar bands to pedicle screw constructs provides an effective means of enhancing axial rotation correction in AIS surgery without increasing the risk of complications. Both constructs yielded comparable outcomes in coronal and sagittal plane correction, suggesting that sublaminar bands can be a useful tool in the armamentarium of spine surgeons managing complex scoliotic deformities.

## Data Availability

The datasets generated and analysed during the current study are available from the corresponding author upon reasonable request.

## Abbreviations

AIS: Adolescent idiopathic scoliosis
PSF: Posterior spinal fusion
BMI: Body mass index
AP: Anteroposterior
LL: Lumbar lordosis
TK: Thoracic kyphosis
SPSS: Statistical package for the social sciences
DICOM: Digital Imaging and communications in medicine

## References

[1] Shakil H, Iqbal ZA, Al-Ghadir AH. Scoliosis: review of types of curves, etiological theories and Conservative treatment. J Back Musculoskelet Rehabil. 2014;27(2):111–5. 24284269 10.3233/BMR-130438

[2] Bridwell KH. Surgical treatment of idiopathic adolescent scoliosis. Spine (Phila Pa 1976). 1999;24(24):2607–16. 10635524 10.1097/00007632-199912150-00008

[3] Grabala P, Gregorczyk J, Fani N, Galgano MA, Grabala M. Surgical treatment strategies for severe and neglected spinal deformities in children and adolescents without the use of radical Three-Column osteotomies. J Clin Med. 2024;13(16):4824. 10.3390/jcm13164824 PMC11355333 39200966

[4] Pesenti S, Ghailane S, Varghese JJ, Ollivier M, Peltier E, Choufani E, et al. Bone substitutes in adolescent idiopathic scoliosis surgery using sublaminar bands: is it useful? A case-control study. Int Orthop. 2017;41(10):2083–90. 28540414 10.1007/s00264-017-3512-4

[5] Zaher A, El Youssef K, Decourtivron B, Bergerault F, Bonnard C, Odent T. Efficacy of polyester bands placed under the transverse vertebral process for the correction of adolescent idiopathic scoliosis: A case series of 105 patients with a minimum of 24 months follow-up. Eur Spine J. 2021;30(7):1959–64. 33881643 10.1007/s00586-021-06841-0

[6] Weiss HR. Measurement of vertebral rotation: perdriolle versus Raimondi. Eur Spine J. 1995;4(1):34–8. 7749905 10.1007/BF00298416

[7] Ilharreborde B, Sebag G, Skalli W, Mazda K. Adolescent idiopathic scoliosis treated with posteromedial translation: radiologic evaluation with a 3D low-dose system. Eur Spine J. 2013;22(11):2382–91. 23580058 10.1007/s00586-013-2776-7 PMC3886523

[8] Kilicaslan OF, Akalin S, Tokgoz MA, Cetin H, Etli I. Comparison of pedicle screws versus hybrid fixation with sublaminar polyester bands in the treatment of neuromuscular scoliosis. World Neurosurg. 2021;151:e672–81. 33940277 10.1016/j.wneu.2021.04.097

[9] Canavese F, Samba A. Sublaminar polyester bands for the correction of idiopathic and neuromuscular scoliosis. Ann Transl Med. 2020;8(2):32. 32055623 10.21037/atm.2019.08.109 PMC6995906

[10] Cinnella P, Rava A, Mahagna AA, Fusini F, Masse A, Girardo M. Over 70 degrees thoracic idiopathic scoliosis: results with screws or hybrid constructs. J Craniovertebr Junction Spine. 2019;10(2):108–13. 31404131 10.4103/jcvjs.JCVJS_39_19 PMC6652256

[11] Noe MC, Link RC, Warren JR, Etebari CV, Whitmire MH, Anderson JT, et al. Three-dimensional deformity correction in adolescent idiopathic scoliosis patients: what are the benefits of hybrid apical sublaminar bands versus all-pedicle screws? J Pediatr Orthop B. 2025;34(4):367–74. 10.1097/BPB.0000000000001204 39229888

[12] Polirsztok E, Gavaret M, Gsell T, Suprano I, Choufani E, Bollini G, et al. Sublaminar bands: are they safe? Eur Spine J. 2015;24(7):1441–9. 25291975 10.1007/s00586-014-3594-2

[13] Gallazzi E, Pallotta LM, La Maida GA, Luca A, Bassani T, Brayda-Bruno M. Is posteromedial translation with sublaminar bands effective in correcting axial rotation in adolescent idiopathic scoliosis surgery? A 3D reconstruction study. Eur Spine J. 2023;32(1):202–9. 36372841 10.1007/s00586-022-07449-8

[14] Sikora-Klak J, Upasani VV, Ilharreborde B, Cross M, Bastrom TP, Mazda K, et al. Three-dimensional analysis of spinal deformity correction in adolescent idiopathic scoliosis: comparison of two distinct techniques. Childs Nerv Syst. 2021;37(2):555–60. 32839853 10.1007/s00381-020-04868-0

[15] La Maida GA, Peroni DR, Ferraro M, Della Valle A, Vitali C, Misaggi B. Apical vertebral derotation and translation (AVDT) for adolescent idiopathic scoliosis using screws and sublaminar bands: a safer concept for deformity correction. Eur Spine J. 2018;27(Suppl 2):157–64. 29846809 10.1007/s00586-018-5626-9

[16] Rosenfeld S, Kenney S, Rebich E. Sublaminar polyester band fixation construct in the treatment of neuromuscular scoliosis. J Child Orthop. 2019;13(4):393–8. 31489045 10.1302/1863-2548.13.190059 PMC6701441

[17] Hirsch C, Ilharreborde B, Fournier J, Mazda K, Bonnard C. Adolescent idiopathic scoliosis correction achieved by posteromedial translation using polyester bands: A comparative study of subtransverse process versus sublaminar fixation. Orthop Traumatol Surg Res. 2014;100(7):791–5. 25442051 10.1016/j.otsr.2014.07.019

[18] Kumar V, Vatkar AJ, Raj DV, Barik S, Richa R. Does hybrid instrumentation using sublaminar bands give comparable results to all pedicle screw constructs in surgical correction of adolescent idiopathic scoliosis?? A systematic review and Meta-Analysis of current evidence. Turk Neurosurg. 2025;35(2):189–201. 40129196 10.5137/1019-5149.JTN.46366-24.3

[19] Palmisani M, Dema E, Cervellati S, Palmisani R. Hybrid constructs pedicle screw with apical sublaminar bands versus pedicle screws only for surgical correction of adolescent idiopathic scoliosis. Eur Spine J. 2018;27(Suppl 2):150–6. 29774412 10.1007/s00586-018-5625-x

[20] Pasha S, Ilharreborde B, Baldwin K. Sagittal spinopelvic alignment after posterior spinal fusion in adolescent idiopathic scoliosis: A systematic review and Meta-analysis. Spine (Phila Pa 1976). 2019;44(1):41–52. 29889799 10.1097/BRS.0000000000002736

